# Deep Q-Learning-Based Transmission Power Control of a High Altitude Platform Station with Spectrum Sharing

**DOI:** 10.3390/s22041630

**Published:** 2022-02-19

**Authors:** Seongjun Jo, Wooyeol Yang, Haing Kun Choi, Eonsu Noh, Han-Shin Jo, Jaedon Park

**Affiliations:** 1 Department of Electronic Engineering, Hanbat National University, Daejeon 34158, Korea; 30211176@edu.hanbat.ac.kr (S.J.); 30211173@edu.hanbat.ac.kr (W.Y.); 2 TnB Radio Tech., Seoul 08504, Korea; radioeng@tnbrt.com; 3 Agency for Defense Development, Daejeon 34186, Korea; nes@add.re.kr

**Keywords:** Deep Q-learning (DQL), Double Deep Q-learning (DDQL), dynamic spectrum sharing, High Altitude Platform Station (HAPS), cellular communications, power control, interference management

## Abstract

A High Altitude Platform Station (HAPS) can facilitate high-speed data communication over wide areas using high-power line-of-sight communication; however, it can significantly interfere with existing systems. Given spectrum sharing with existing systems, the HAPS transmission power must be adjusted to satisfy the interference requirement for incumbent protection. However, excessive transmission power reduction can lead to severe degradation of the HAPS coverage. To solve this problem, we propose a multi-agent Deep Q-learning (DQL)-based transmission power control algorithm to minimize the outage probability of the HAPS downlink while satisfying the interference requirement of an interfered system. In addition, a double DQL (DDQL) is developed to prevent the potential risk of action-value overestimation from the DQL. With a proper state, reward, and training process, all agents cooperatively learn a power control policy for achieving a near-optimal solution. The proposed DQL power control algorithm performs equal or close to the optimal exhaustive search algorithm for varying positions of the interfered system. The proposed DQL and DDQL power control yields the same performance, which indicates that the actional value overestimation does not adversely affect the quality of the learned policy.

## 1. Introduction

A High Altitude Platform Station (HAPS) is a network node operating in the stratosphere at an altitude of approximately 20 km. The International Telecommunication Union (ITU) defines a HAPS in Article 1.66A as “A station on an object at an altitude of 20 to 50 km and a specified, nominal, fixed point relative to the Earth”. Various studies have been performed on HAPS in recent years, and the commercial applications of HAPS have significantly increased [1]. In addition, the HAPS has potential as a significant component of wireless network architectures [2]. It is also an essential component of next-generation wireless networks, with considerable potential as a wireless access platform for future wireless communication systems [3,4,5].

Because the HAPS is located at high altitudes ranging from 20 to 50 km, the HAPS-to-ground propagation generally experiences lower path loss and a higher line-of-sight probability than typical ground-to-ground propagation. Thus, the HAPS can provide a high data rate for wide coverage; however, it is likely to interfere with various other terrestrial services, e.g., fixed, mobile, and radiolocation. The World Radiocommunication Conference 2019 (WRC-19) adopted a HAPS as the IMT Base Station (HIBS) in the frequency bands below 2.7 GHz previously identified for IMT by Resolution 247 [6], which addresses the potential interference of HAPS with an existing service. In such a situation, if the existing service is not safe from HAPS interference, the two systems cannot coexist. Therefore, the HAPS transmitter is requested to reduce its transmission power to satisfy the interference–to–noise ratio (INR) requirement for protecting the receiver of the existing service. However, if the HAPS transmission power is excessively reduced, the signal–to–interference–plus–noise ratio (SINR) of the HAPS downlink decreases; thus, the outage probability may exceed the desired level. Herein, a HAPS transmission power control algorithm is proposed that aims to minimize the outage probability of the HAPS downlink while satisfying the INR requirement for protecting incumbents.

### 1.1. Related Works

Studies have been performed on improving the performance of HAPS. In [7], resource allocation for an Orthogonal Frequency Division Multiple Access (OFDMA)-based HAPS system that uses multicasting in the downlink to maximize the number of user terminals by maximizing the radio resources was studied. The authors of [8] proposed a wireless channel allocation algorithm for a HAPS 5G massive multiple-input multiple-output (MIMO) communication system based on reinforcement learning. Combining Q-learning and backpropagation neural networks allows the algorithm to learn intelligently for varying channel load and block conditions. In [9], a criterion for determining the minimum distance in a mobile user access system was derived, and a channel allocation approach based on predicted changes in the number of users and the call volume was proposed.

Additionally, spectrum sharing studies on HAPS have been performed. In [10], a spectrum sharing study was conducted to share a fixed service using a HAPS with other services in the 31/28-GHz band. Interference mitigation techniques were introduced, e.g., increasing the minimum operational elevation angle or improving the antenna radiation pattern to facilitate sharing with other services. In addition, the possibility of dynamic channel allocation was analyzed. In [11], sharing between a HAPS and a fixed service in the 5.8-GHz band was investigated using a coexistence methodology based on a spectrum emission mask.

In contrast to previous studies in which HAPS communication improvement and spectrum sharing were dealt with separately, in the present study, a combination of spectrum sharing with other systems and HAPS downlink coverage improvement is considered. In this regard, this study is more advanced than previous HAPS-related studies.

Deep Q-learning (DQL) is a reinforcement learning algorithm that applies deep neural networks to reinforcement learning to solve complex problems in the real world. DQL is widely used in various fields, including UAV, drone, and HAPS. In [12], the optimal UAV-BS trajectory was presented using a DQL for optimal placement of UAVs, and the author of [13] used a DQL to determine the optimal link between two UAV nodes. In [14], a DQL is used to find the optimal flight parameters for the collision-free trajectory of the UAV. In [15], two-hop communication was considered to optimize the drone base station trajectory and improve network performance, and a DQL was used to solve the joint two-hop communication scenario. In [16], a DQL was used for multiple-HAPS coordination for communications area coverage. A Double Deep Q-learning (DDQL) is an algorithm developed to prevent the overestimation of a DQL and shows better performance than the DQL in various fields [17].

### 1.2. Contributions

The contributions of the present study are as follows. (1) For the first time, a multiagent DQL was used to improve the HAPS outage performance and solve the problem of spectrum sharing with existing services. (2) We defined the power control optimization problem to minimize the outage probability of the HAPS downlink under the interference constraint for protecting the existing system. The state and reward for the training agent were designed to consider the objective function and constraints of the optimization problem. (3) Because the HAPS has a multicell structure, the number of power combinations increases exponentially as the number of cells ($N_{cell}$) and power levels increase linearly. Thus, the optimal exhaustive search method requires an impractically long computation time to solve the multicell power optimization problem. The proposed DQL algorithm performs comparably to an optimal exhaustive search with a feasible computation time. (4) Even for varying positions of the interfered system, the proposed DQL produces a proper power control policy, maintaining stable performance. (5) Comparing the proposed DQL algorithm with the DDQL algorithm shows no performance degradation due to overestimation in the proposed DQL. The remainder of this paper is organized as follows.

Section 2 presents the system model, including the system deployment model, HAPS model, interfered system model, and path loss model. In Section 3, the downlink SINR and INR are calculated. In Section 4, a DQL-based HAPS power control algorithm is proposed. Section 5 presents the simulation results, and Section 6 concludes the paper.

## 2. System Model

### 2.1. System Deployment Model

**Figure 1:**
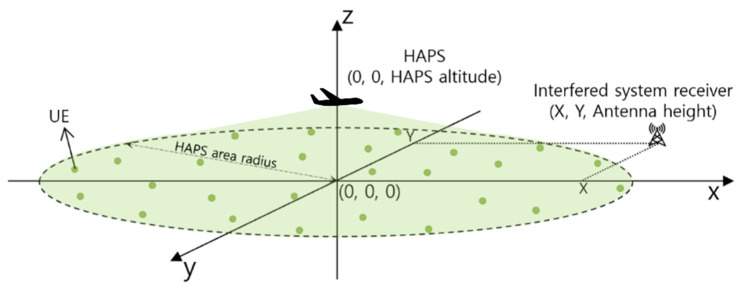
System deployment model.

HAPS communication networks are assumed to consist of a single HAPS, multiple ground user equipment (*UE*) devices (referred to as *UE*s hereinafter), and a ground interfered receiver. The HAPS, *UE*, and interfered receiver are distributed in the three-dimensional Cartesian coordinate system, as shown in Figure 1. The coordinates of the HAPS antenna and the interfered receiver antenna are (0, 0, $h_{HAPS}$) and (X, Y, $h_{V}$), respectively. The $N_{UE}$ *UE* devices with an antenna height of $h_{UE}$ are uniformly distributed within the circular HAPS area.

### 2.2. HAPS Model

**Figure 2:**
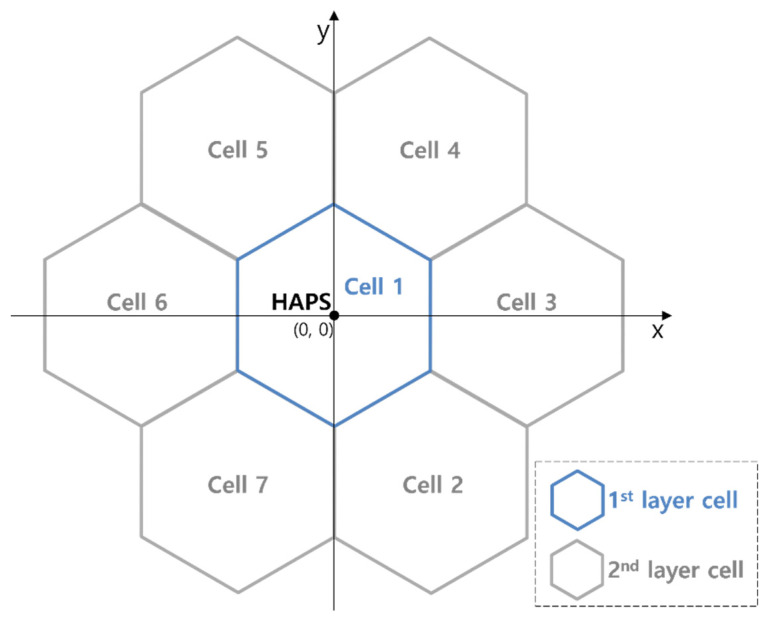
HAPS seven-cell layout.

**Figure 3:**
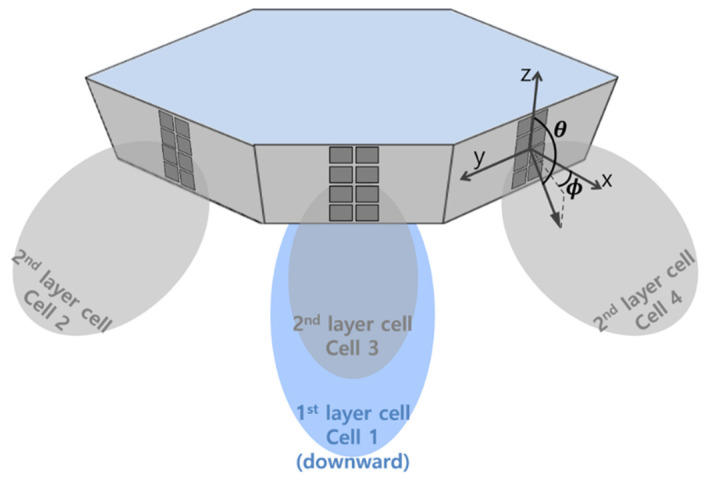
Typical antenna structure for multi-cell HAPS communication.

We modeled the HAPS cell deployment and system parameters with reference to the working document for a HAPS coexistence study performed in preparation for WRC-23 [18]. As shown in Figure 2, a single HAPS serves multiple cells that consist of one 1st layer cell denoted as Cell_1 and six 2nd layer cells denoted as Cell_2 to Cell_7. The six cells of the 2nd layer are arranged at intervals of 60° in the horizontal direction. Figure 3 presents a typical HAPS antenna design for seven-cell structures [4], where seven phased-array antennas conduct beamforming toward the ground to form seven cells, as shown in Figure 2. The 1st layer cell has an antenna tilt of 90°, i.e., perpendicular to the ground; the 2nd layer cell has an antenna tilt of 23°.

The antenna pattern of the HAPS was designed using the antenna gain formula presented in Recommendation ITU-R M.2101 [19]. The transmitting antenna gain is calculated as the sum of the gain of a single element and the beamforming gain of a multi-antenna array. The single element antenna gain is determined by the azimuth angle (ϕ) and the elevation angle (θ) between the transmitter and receiver and is calculated as follows:

(1) $$A_{E}\left(\phi ,\theta \right)=G_{E,max}-min{\left\{-\left[A_{E,H}\left(\phi \right)+A_{E,v}\left(\theta \right)\right],A_{m}\right\}}_{\;},$$

where $G_{E,max}$ represents the maximum antenna gain of a single element, $A_{E,H}\left(\phi \right)$ represents the horizontal radiation pattern calculated using Equation (2), and $A_{E,v}\left(\theta \right)$ represents the vertical radiation pattern calculated using Equation (3).

(2) $$A_{E,H}\left(\phi \right)=-min\left[12{\left(\frac{\phi }{\phi_{3\mathrm{dB}}}\right)}^{2},A_{m}\right]$$

Here, $\phi_{3\mathrm{dB}}$ represents the horizontal 3 dB beamwidth of a single element, and $A_{m}$ represents the front-to-back ratio.

(3) $$A_{E,V}\left(\theta \right)=-min\left[12{\left(\frac{\theta -90}{\theta_{3\mathrm{dB}}}\right)}^{2},SLA_{v}\right]$$

Here, $\theta_{3\mathrm{dB}}$ represents the vertical 3 dB bandwidth of a single element, and $SLA_{v}$ represents the front-to-back ratio.

The transmitting antenna gain of the HAPS is calculated using the antenna arrangement and spacing, as well as the target beamforming direction. The gain for beam *i* is calculated as follows:

(4) $$A_{A,Beami}\left(\theta ,\phi \right)=A_{E}\left(\theta ,\phi \right)+10\log_{10}\left({\left|\sum_{m=1}^{N_{H}}\;\sum_{n=1}^{N_{V}}w_{i,n,m}\cdot v_{n,m}\right|}^{2}\right),$$

where $N_{H}$ and $N_{V}$ represent the number of antennas in the horizontal and vertical directions, respectively. $v_{n,m}$ is the superposition vector that overlaps the beams of the antenna elements, which is calculated using Equation (5), and $w_{i,n,m}$ is the weight that directs the antenna element in the beamforming direction, which is calculated using Equation (6).

(5) $$\begin{array}{c} n=1,2,\ldots N_{V};m=1,2,\ldots N_{H}\\ v_{n,m}=exp\left(\sqrt{-1}\cdot 2\pi \left(\left(n-1\right)\cdot \frac{d_{V}}{\lambda }\cdot cos\left(\theta \right)+\left(m-1\right)\cdot \frac{d_{H}}{\lambda }\cdot sin(\theta )\cdot sin(\phi )\right)\right) \end{array}$$

Here, $d_{H}\;\mathrm{and}\;d_{V}$ represent the intervals between the horizontal and vertical antenna arrays, respectively, and λ represents the wavelength.

(6) $$\begin{array}{cc} w_{i,n,m}=\frac{1}{\sqrt{N_{H}N_{V}}} & exp(\sqrt{-1}\\ & \cdot 2\pi \left(\left(n-1\right)\cdot \frac{d_{V}}{\lambda }\cdot sin\left(\theta_{i,etilt}\right)-\left(m-1\right)\cdot \frac{d_{H}}{\lambda }\cdot cos(\theta_{i,etilt}\right)\\ & \cdot sin(\phi_{i,escan}))) \end{array}$$

Here, $\phi_{i,escan}$ and $\theta_{i,etilt}$ represent the ϕ and θ of the main beam direction, respectively.

**Figure 4:**
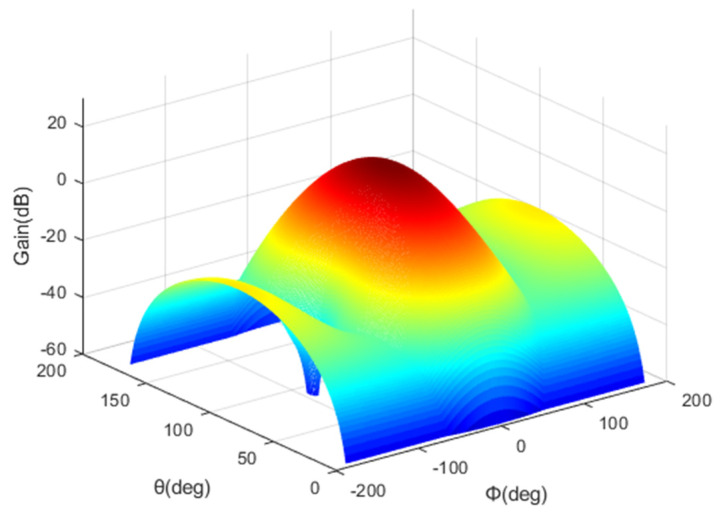
1st layer cell antenna pattern.

**Figure 5:**
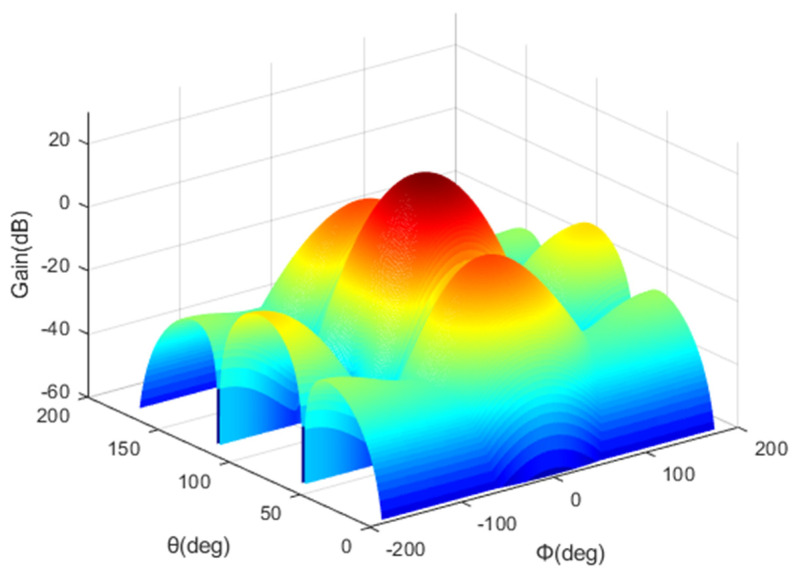
2nd layer cell antenna pattern.

The 1st layer cell of the HAPS uses a 2 × 2 antenna array, and the 2nd layer cell uses a 4 × 2 antenna array. Figure 4 shows the antenna pattern of the 1st layer cell, and Figure 5 shows the antenna pattern of the 2nd layer cell.

### 2.3. Interfered System Model

Various interfered systems, e.g., fixed, mobile, and radiolocation services, can be considered for the interference scenario involving a HAPS. We adopted a ground IMT base station (BS) for the interfered system, referring to the potential interference scenario [6]. The antenna pattern of the interfered system was applied by referring to Recommendation ITU-R F.1336 [20]. The receiving antenna gain is calculated as follows:

(7) $$G\left(\phi ,\theta \right)=G_{0}+G_{hr}\left(x_{h}\right)+R\cdot G_{vr}\left(x_{v}\right),$$

where $G_{0}$ represents the maximum gain in the azimuth plane; $G_{hr}\left(x_{h}\right)$ represents the relative reference antenna gain in the azimuth plane in the normalized direction of ($x_{h}$, 0), which is calculated using Equation (8); and $G_{vr}\left(x_{v}\right)$ represents the relative reference antenna gain in the elevation plane in the normalized direction of (0, $x_{v}$), which is calculated using Equation (9). R represents the horizontal gain compression ratio when the azimuth angle is shifted from 0° to ϕ, which is calculated using Equation (10).

(8) $$\begin{array}{c} G_{hr}\left(x_{h}\right)=-12x_{h}^{2}\;\;\;\;\;\;\;\;\;\;\;\;\;\;\;\;\;\;\;\;\;\;\;\;\;\;\;\;\;\;\;for\;x_{h}\leq 0.5_{\;}\\ G_{hr}\left(x_{h}\right)=-12x_{h}^{\left(2-k_{h}\right)}-\lambda_{\mathrm{kh}}\;\;\;\;\;\;\;\;\;\;\;\;\;for\;0.5<x_{h}\\ G_{hr}\left(x_{h}\right)\geq G_{180} \end{array}$$

(9) $$\begin{array}{c} G_{\mathrm{vr}}\left(x_{v}\right)=-12x_{v}^{2}\;\;\;\;\;\;\;\;\;\;\;\;\;\;\;\;\;\;\;\;\;\;\;\;\;\;\;\;\;\;\;\;\;\;\;\;\;\;\;\;\;\;\;\;\;\;\;\;\;\;for\;x_{v}<x_{k}\\ G_{\mathrm{vr}}\left(x_{v}\right)=-15+10\log(x_{v}^{-1.5}+k_{v})\;\;\;\;\;\;\;\;\;\;\;\;\;\;for\;x_{k}\leq x_{v}<4\\ G_{\mathrm{vr}}\left(x_{v}\right)=-\lambda_{\mathrm{kv}}-3-C\log(x_{v})\;\;\;\;\;\;\;\;\;\;\;\;\;\;\;\;\;\;\;\;\;for\;4\leq x_{v}<90˚/\theta_{3}\\ G_{\mathrm{vr}}\left(x_{v}\right)=G_{180}\;\;\;\;\;\;\;\;\;\;\;\;\;\;\;\;\;\;\;\;\;\;\;\;\;\;\;\;\;\;\;\;\;\;\;\;\;\;\;\;\;\;\;\;\;\;\;\;\;\;\;\;\;for\;x_{v}\geq 90˚/\theta_{3} \end{array}$$

(10) $$R=\frac{G_{hr}\left(x_{h}\right)-G_{hr}\left(180{}^{\circ}/\phi_{3}\right)}{G_{hr}\left(0\right)-G_{hr}\left(180{}^{\circ}/\phi_{3}\right)}$$

Here, $x_{h}$ and $\lambda_{kh}$ are given by Equations (11) and (12), respectively; $\phi_{3}$ represents the 3 dB beamwidth in the azimuth plane; and $k_{h}$ is an azimuth pattern adjustment factor based on the leaked power. The relative minimum gain $G_{180}$ was calculated using Equation (13).

(11) $$x_{h}=\left|\phi_{\;}\right|/\phi_{3}$$

(12) $$\lambda_{\mathrm{kh}}=3\;\left(1-0.5_{\;}^{-k_{h}}\right)$$

(13) $$G_{180}=-15+10\log\left(1+8k_{a}\right)-15\log\left(\frac{180{}^{\circ}}{\theta_{3}}\right)\;$$

**Figure 6:**
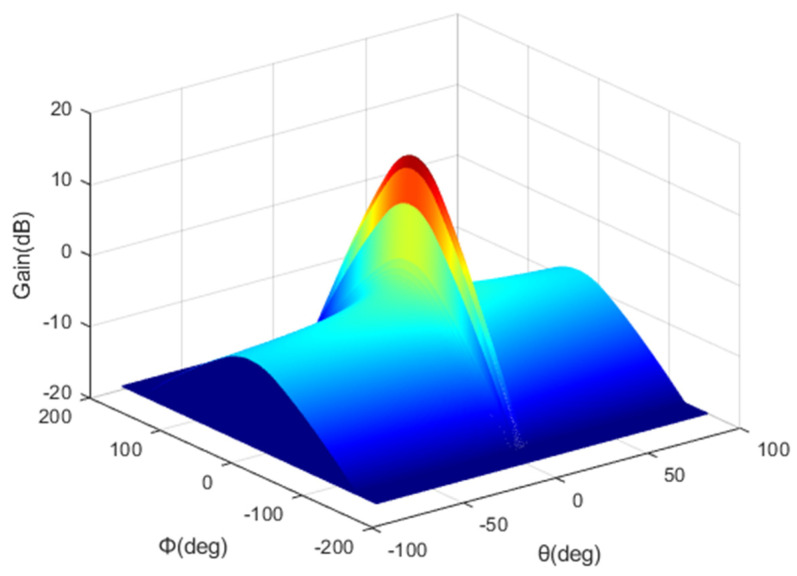
Interfered system antenna pattern.

Returning to Equation (9), $x_{v}$ is given by Equation (14), and the 3-dB beamwidth in the elevation plane $\theta_{3}$ is calculated using Equation (15), where $G_{0}$ represents the maximum gain in the azimuth plane. In addition, $x_{k}$ is calculated using Equation (16), where $k_{v}$ is an elevation pattern adjustment factor based on the leaked power. $\lambda_{\mathrm{kv}}$ was calculated using Equation (17), and the attenuation inclination factor C was calculated using Equation (18). Figure 6 shows the antenna pattern of the interfered system calculated using Equation (7), which is the pattern for a typical terrestrial BS with a broad beamwidth in the azimuth plane but a narrow beamwidth in the elevation plane.

(14) $$x_{v}=\left|\theta_{\;}\right|/\theta_{3}$$

(15) $$\theta_{3}=107.6\times 10^{-0.1G_{0}}$$

(16) $$x_{k}=\sqrt{1.33-0.33k_{v}}$$

(17) $$\lambda_{\mathrm{kv}}=12-C\log\left(4\right)-10\log\left(4^{-1.5}+k_{v}\right)$$

(18) $$C=\frac{10\log\left(\frac{{\left(\frac{180{}^{\circ}}{\theta_{3}}\right)}^{1.5}\cdot \;\left(4^{-1.5}+k_{v}\right)}{1+8k_{p}}\right)}{\log\left(\frac{22.5{}^{\circ}}{\theta_{3}}\right)}$$

### 2.4. Path Loss Model

The path loss model of Recommendation ITU-R P.619 [21] was applied to the working document for the HAPS coexistence study performed in preparation for WRC-23 [22]. The total path loss that occurs when the HAPS signal reaches the *UE* and the IMT BS is expressed as follows:

(19) $$L_{p}=FSL+A_{xp}+A_{g}+A_{bs},$$

where FSL represents the free-space path loss calculated using Equation (20), which occurs in a straight path from a transmitting antenna to a receiving antenna in a vacuum state, and $A_{xp}$ is assumed to be 3 dB for depolarization attenuation. $A_{g}$ represents the attenuation loss due to atmospheric gases. $A_{bs}$ represents the resistive loss due to the spread of the antenna beam as the beam spreads attenuation. $A_{g}$ and $A_{bs}$ were calculated using the formulae in P.619.

(20) FSL=92.45+20log(f·d)

Here, f represents the carrier frequency (in GHz), and d represents the distance (in km) between the transmitter and receiver.

## 3. Calculation of Downlink SINR and INR

### 3.1. Calculation of Downlink SINR

The signal received by the *UE* from the HAPS transmission for the ith cell (Cell_i) is calculated as follows:

(21) $$S_{Cell\_i}=P_{Cell\_i}+G_{Cell\_i}+G_{p}+G_{r,UE}-L_{p}-L_{ohm},$$

where $P_{Cell\_i}$ represents the HAPS transmission power for Cell_i, $G_{Cell\_i}$ represents the transmitting antenna gain of Cell_i, $G_{p}$ represents the polarization gain, $G_{r,UE}$ represents the receiving antenna gain, and $L_{ohm}$ represents the ohmic loss. The *UE* receives signals from all $N_{cell}$ cells and considers the remaining signals (except for the strongest Cell j signal) as interference. Equation (22) is used to calculate the signal and interference, and the receiver noise is calculated using Equation (23).

(22) $$j=\underset{i}{\mathrm{argmax}}S_{Cell\_i}\;S_{HAPS}=S_{Cell\_j}\;I_{HAPS,UE}=10\log(\sum_{\begin{array}{c} i=1\;\\ i\neq j \end{array}}^{N_{cell}}10^{\frac{S_{Cell\_i}}{10}})$$

(23) $$N=10\log\left(k\times T\times BW\right)+N_{f}\;$$

Here, k and T represent the Boltzmann constant and noise temperature, respectively, and BW represents the channel bandwidth. $N_{f}$ represents the noise figure. Finally, the downlink SINR is calculated as follows:

(24) $$\eta =10\log\left(\frac{10^{\frac{S_{HAPS}}{10}}}{10^{\frac{I_{HAPS,UE}}{10}}+10^{\frac{N}{10}}}\right).\;$$

### 3.2. Calculation of INR

The interference power received by the interfered receiver from the HAPS transmitter servicing Cell i is calculated as follows:

(25) $$I_{Cell\_i}=P_{Cell\_i}+G_{Cell\_i}+G_{p}+G_{r,V}-L_{p}-L_{ohm},$$

where $G_{r,V}$ represents the antenna gain of the interfered receiver. The aggregated interference power at the interfered receiver is calculated as follows:

(26) $$I_{HAPS,V}=10\log\left(\overset{N_{cell}}{\underset{i=1\;}{\sum }}10^{\frac{I_{Cell\_i}}{10}}\right).$$

Finally, after converting the aggregated interference into INR form in accordance with Equation (27) and comparing it with the protection criteria ($INR_{th}$) of the interfered receiver, it is possible to check whether the interfered receiver is protected from the interference of the HAPS.

(27) $$INR=I_{HAPS,V}-N$$

## 4. DQL-Based HAPS Transmission Power Control Algorithm

### 4.1. Problem Formulation

To satisfy the $INR_{th}$ of the interfered system, the transmission power of the HAPS must be reduced. However, as the power of the HAPS is reduced, the η of the *UE* decreases, and the outage probability $P_{out}$ increases. Thus, the objective of this study was to find a HAPS transmission power set for each cell, i.e., $P=\left\{P_{Cell\_i}|i=1,\cdots ,N_{cell}\right\}$, that satisfies the $INR_{th}$ of the interfered system while minimizing $P_{out}$. The optimization problem of the HAPS transmission power can be formulated as follows:

(28) minPPout=NUE,o(P)NUEs.t.C1:  INR≤INRth C2:   Pmin≤PCelli≤Pmax  ∀i∈{1,⋯,Ncell},

where $N_{UE,o}\left(P\right)$ represents the number of UEs that do not satisfy the minimum required SINR $\eta_{o}$ for a given HAPS transmission power set P.

### 4.2. Proposed Algorithm

To control the HAPS transmission power, it is necessary to independently determine the power level of each cell. Accordingly, the total number of HAPS transmission power sets increases exponentially to $N_{p}^{N_{cell}}$ as the number of selectable powers $N_{p}$ increases linearly. Although an exhaustive search algorithm can be used to find optimal solutions, this incurs excessive complexity and a long computation time. To solve this problem, we propose a DQL-based power optimization algorithm that can find a near-optimal P with low complexity. In the proposed DQL model, each agent functions as the power controller of a cell; accordingly, the number of agents is $N_{cell}$.

The agent—the subject of learning—learns a deep neural network called Deep Q Network (DQN) and selects an action using this network. DQL is an improved Q-learning method. Q-learning is a method for selecting the best action in a specific state through the Q-table of a state-action pair. As the state–action space grows in Q-learning, creating a Q-table and finding the best policy become highly complex. In addition, the use of Q-learning is limited because learning in the Q-table format becomes more complex when multiple agents are used. In contrast, a DQL is a promising way to solve the curse of dimensionality by approximating a Q function using a deep neural network instead of a Q-table. The proposed algorithm uses a method in which each agent learns a policy based on its observation and action while treating all other agents as part of the environment to solve the multiple-agent problem.

The basic DQL parameters (state, action, and reward) are presented below. Each agent learns the policy independently using the training data at each timestep t. The state space of the m^th^ agent comprises a set of $(N_{cell}-1$) interferences that the agent provides to UEs located at the centers of other cells and the agent’s interference to the interfered receiver, which is expressed as

(29) $$S_{t}=\left\{I_{v},\left\{I_{UE\_i}|i=1,\cdots ,N_{cell},\;\;and\;\;i\neq m\right\}\right\}.$$

Two power sets configure the action space of an agent: $A_{1}=\left\{29,\;31,\;33,\;35,\;37\right\}$ and $A_{2}=\left\{26,\;28,\;30,\;32,\;34\right\}$ (unit: dBm). The agent of Cell_1 in the 1st layer cell selects an action from $A_{1}$, and the agents of the 2nd layer cell select an action from $A_{2}$. All agent actions are initialized to the minimum power value to minimize the interference to the interfered receiver at the beginning of the learning process. The reward is calculated as follows. First, because the interfered receiver must be safe from HAPS interference, an agent receives a fixed $r_{t}$ of −100 (deficient value) for $INR>INR_{th}$. In contrast, for $INR\leq INR_{th}$, an agent receives $r_{t}$ computed according to the lower 5% downlink SINR of each cell $\left\{{\hat{\eta }}_{i}|i=1,\;2,\;\cdots ,\;N_{cell}\right\}$ and the required SINR $\eta_{o}$. The reward can be expressed as

(30) $$r_{t}=\{\begin{array}{cc} r_{1,\;t}+r_{2,\;t} & for\;INR\leq INR_{th}\\ r_{t}=-100 & otherwise, \end{array},$$

where

(31) $$r_{1,t}=10\cdot \left(\sum \left({\hat{\eta }}_{i}-\eta_{o}\right)\right)\;\;\;for\;{\hat{\eta }}_{i}\geq \eta_{o}\;r_{2,t}=\sum \left({\hat{\eta }}_{i}+\eta_{o}\right)\;\;\;\;\;\;\;\;\;\;\;\;\;for\;{\hat{\eta }}_{i}<\eta_{o}.$$

**Figure 7:**
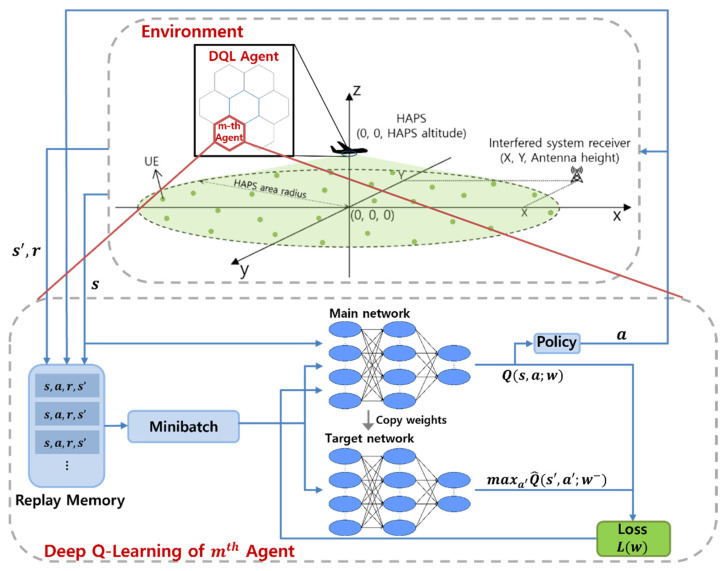
DQL-based HAPS power control architecture.

Figure 7 shows the structure of the proposed DQL-based HAPS transmission power control algorithm. Each agent learns its DQN, and one DQN consists of the main network, target network, and replay memory. The main network estimates the *Q*-value Q(s,a;w) corresponding to the state–action pair through a deep neural network with a weight w. The main network consists of an input layer composed of seven neurons, a hidden layer consisting of 24 neurons, and an output layer consisting of five neurons. It is a fully connected network. w is updated every t in the direction that minimizes the loss function $L\left(w\right)=E\left[{\left(y_{j}-Q\left(s,a;w\right)\right)}^{2}\right]$. The target network calculates the target value $y_{j}=r_{j}+\gamma \underset{a^{\prime }}{\max}\hat{Q}\left(s^{\prime },a^{\prime };w^{-}\right)$, where γ is the discount factor; $s^{\prime }$ and $a^{\prime }$ denotes the state and action, respectively, in the next step; and $\hat{Q}\left(s^{\prime },a^{\prime };w^{-}\right)$ is the *Q*-value estimated through the target network with weight $w^{-}$. The agent’s transition tuple $\left(s_{t},\;a_{t},\;r_{t},\;s_{t+1}\right)$ is piled in the replay memory, from which a minibatch (size of 512 tuples) are randomly sampled at each step. The minibatch data are used to compute the target value $y_{j}$. In a DQL, learning is stabilized, and the learning performance is improved through replay memory and a separate target network [23].

Algorithm 1 describes the proposed DQL-based HAPS transmission power control algorithm. For DQN training, N was set as 100,000, and the minibatch size was set as 512. M was set as 500, and T was set as 10. The Adam optimizer was used to minimize L(w), and the learning rate and γ were 0.01 and 0.995, respectively. An ε-greedy policy was used to balance exploration and exploitation; ε was initially set as 1 and was reduced by 0.01 for every episode.


**Algorithm 1.** Training Process for the DQL-Based HAPS Power Control Algorithm
1: Initialize the replay memory D to capacity N2: Initialize the Q-function with random weights w3: Initialize the target $\hat{Q}$-function with the same weights: $w^{-}=w$4: **for** episode = 1, M **do**5:     Initialize action $a_{0}=\underset{a}{\min}A\;$6:     **for** timestep = 1, T **do**7:     **if** t=18:         Calculate $s_{t}$ via Equations (21) and (25)9:     **end if**10:      With probability, select a random action $a_{t}$11:      Otherwise, select $a_{t}=\underset{a}{\arg\max}Q\left(s_{t},a;w\right)$12:      Assign the selected power to the *m*th cell and compute INR and η13:      Observe the reward $r_{t}$ and $s_{t+1}$14:      Store the experience in ($s_{t},\;a_{t},\;r_{t},\;s_{t+1}$) in D15:      Sample a random minibatch of experiences from D16:      Set $y_{j}=r_{j}+\gamma \underset{a^{\prime }}{\max}\hat{Q}\left(s^{\prime },a^{\prime };w^{-}\right)$17:      Perform optimization via L(w) and update w18:      Update the target network $\hat{Q}$ with $w^{-}=w$ every 4 steps19:    **end for**20: 
**end for**




A DDQL is a reinforcement learning algorithm to improve performance degradation due to the overestimation of the DQL. Action-value can be overestimated by the maximization step in line 16 of Algorithm 1. Therefore, the DDQL calculates the target value as $y_{j}=r_{j}+\gamma \hat{Q}\left(s^{\prime },\underset{a^{\prime }}{\mathrm{argmax}}\;Q\left(s^{\prime },a^{\prime };w\right);w^{-}\right)$ to eliminate the maximization step. The DDQL-based HAPS power control algorithm proceeds the same way as Algorithm 1 except for calculating the target value.

## 5. Simulation Results

### 5.1. Simulation Configuration

The simulation was conducted using MATLAB for three positions of the interfered receiver, and the learning order of the agent was randomly set for each t. Subsequently, the simulation proceeded according to Algorithm 1. When all M episodes were finished, the simulation ended, and the set $P_{c}$ composed of the power selected by each agent was calculated as the simulation result. Finally, the performance of the simulation was verified by comparing $P_{c}$ with the optimal power set $P^{*}$ obtained via an exhaustive search algorithm considering all $N_{p}^{N_{cell}}$ cases. The total elapsed time of the DQL and exhaustive search was about 7500 s and 21,000 s, respectively. The total elapsed time of the exhaustive search increased exponentially with the rise of N, but the DQL did not. Therefore, the computational efficiency of the DQL is more remarkable as the number of cells and power levels increase. In this simulation, performance comparison with the DDQL was additionally performed to check performance degradation due to overestimation of the DQL.

We applied the HAPS parameters and interfered system parameters, referring to the working document for the HAPS coexistence study performed in preparation for WRC-23 [18,24]. The simulation parameters of the two systems are presented in Table 1 and Table 2, respectively.

### 5.2. Numerical Analysis

**Figure 8:**
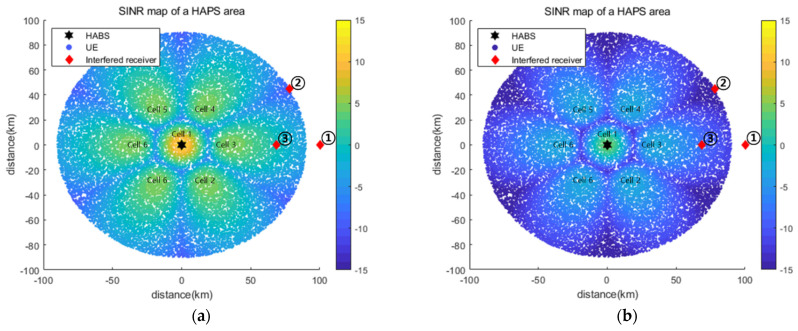
(**a**) SINR map for $P_{max}$; (**b**) SINR map for $P_{min}$.

Figure 8 shows the SINR maps obtained using $P_{max}=\left\{37,\;34,\;34,\;34,\;34,\;34,\;34\right\}$ and $P_{min}=\left\{29,\;26,\;26,\;26,\;26,\;26,\;26\right\}$ for all cells, that is, with no power control. We considered the three positions of the interfered receiver that do not satisfy the $INR_{th}$ of −6 dB for the use of $P_{max}$. In addition, the three locations were designed considering the representative interference power, which can accurately reflect the operating characteristics of the proposed power control algorithm. Interfered receiver ① was located in the main beam direction for Cell_3 and received the highest interference from Cell_3. Therefore, the minimum power use of only Cell_3 satisfied an $INR_{th}$ of −6 dB. Interfered receiver ② was placed on the boundary between Cell_3 and Cell_4 and thus received equal (and the strongest) interference from these two cells. Interfered receiver ③ was located in the main beam direction for Cell_3, as the interfered receiver. However, the minimum power use of only Cell_3 could not satisfy the $INR_{th}$ of −6 dB, and at least one other cell had to use less than the maximum power.

Table 3 presents the INR and $P_{out}$ for $P_{max}$ and $P_{min}$ with varying interfered receiver locations. The results confirm that the $P_{out}$ and INR had a tradeoff relationship. The same $P_{out}$ is shown regardless of the interference receiver position because of the absence of power control. Next, we compared the simulation results of the optimal exhaustive search and the proposed DQL-based power control algorithm for the three positions of the interfered receiver.

#### 5.2.1. Simulation Results for Interfered Receiver ①

**Figure 9:**
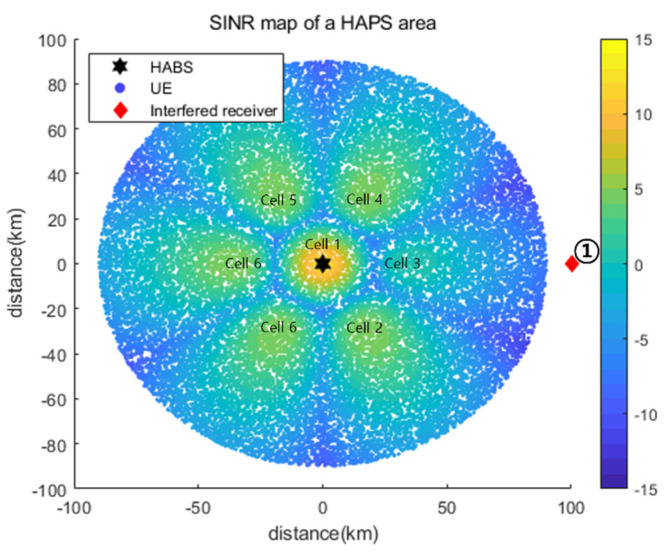
SINR map based on the $P_{c}$ obtained using the proposed DQL-based power control algorithm for interfered receiver ①.

Figure 9 shows the SINR map based on the $P_{c}$ acquired using the proposed DQL-based power control algorithm for interfered receiver ①. Table 4 presents a performance comparison of the $P^{*}$ values obtained via an exhaustive search and $P_{c}$ and a comparison of DQL and DDQL results. As shown, $P_{c}$ was equal to the optimal value $P^{*}$, providing the same $P_{out}$ and INR performance. Because the interfered receiver was located in the azimuth main beam direction of Cell_3, the power of Cell_3 significantly affected the interfered receiver. Even though all other cells used the maximum power, their interference was negligible. Therefore, all the cells except for Cell_3 used the maximum power for minimizing $P_{out}$, as shown in Table 4.

**Figure 10:**
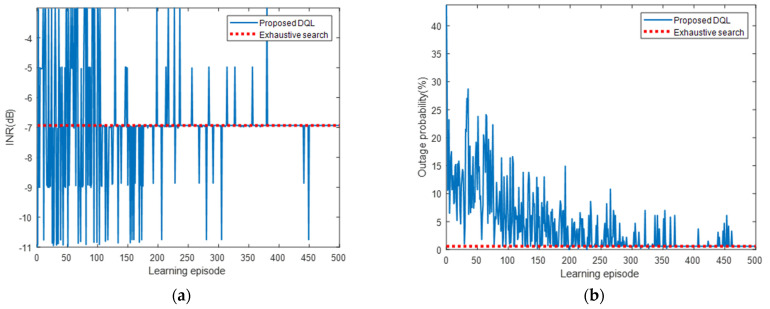
(**a**) INR and (**b**) $p_{out}$ for each learning episode for interfered receiver ①.

**Figure 11:**
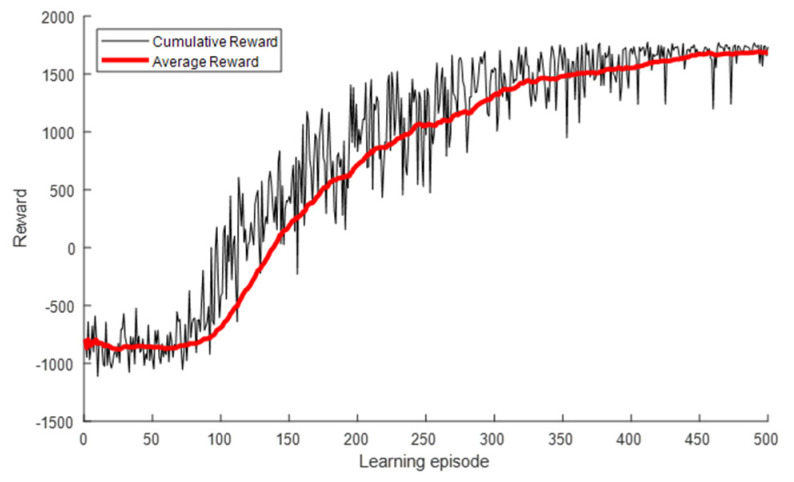
Reward for each learning episode for interfered receiver ①.

Figure 10 presents the INR and $p_{out}$ for each learning episode. As shown, the INR and $p_{out}$ converged to the optimal values of the exhaustive search algorithm as the number of learning episodes increased. The INR started at −11.01 dB, which was the value for the use of $P_{min}$, as shown in Table 3, and converged to the optimal value of −6.93 dB. Similarly, $p_{out}$ started at 43.7% and converged to 0.6%. A large variance due to frequent exploration was observed at the beginning of the learning, but it gradually decreased and converged as the learning progressed. Figure 11 presents the cumulative and average rewards for each learning episode. As shown, the reward rapidly increased and then gradually converged at approximately 300 episodes, indicating that the proposed DQL training process allowed the agent to learn the power control algorithm quickly and stably.

We compared the learning results of the DQL and DDQL. Even when the DDQL is used, the results are the same as in Table 4 and Figure 10 and Figure 11, which shows that the overestimation of the DQL did not occur. As a result, it was confirmed that performance degradation due to overestimation did not happen, and sufficient learning is possible only with DQL.

#### 5.2.2. Simulation Results for Interfered Receiver ②

**Figure 12:**
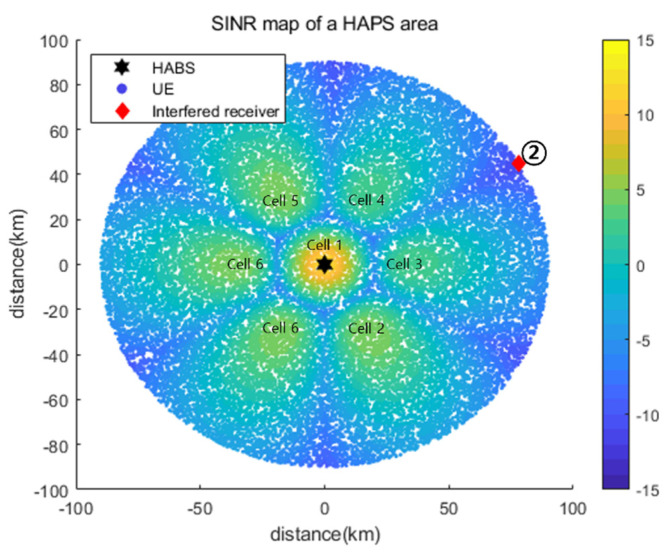
SINR map based on the $P_{c}$ obtained using the proposed DQL-based power control algorithm for the interfered receiver ②.

Figure 12 shows the SINR map based on $P_{c}$ acquired using the proposed DQL-based power control algorithm for interfered receiver ②. Table 5 presents a performance comparison of the $P^{*}$ values obtained via an exhaustive search and $P_{c}$ and a comparison of the DQL and DDQL results. As shown, $P_{c}$ was equal to the optimal value $P^{*}$, providing the same $P_{out}$ and INR performance. The interfered receiver was located on the boundary between Cell_3 and Cell_4 and, thus, received equal (and the strongest) interference from these two cells. In addition, even though all the cells other than Cell_3 and Cell_4 used the maximum power, their interference was marginal. Therefore, in the optimal power control, Cell_3 and Cell_4 reduced the power required to satisfy the $INR_{th}$, whereas all the other cells used the maximum power for minimizing $P_{out}$, as shown in Table 5.

**Figure 13:**
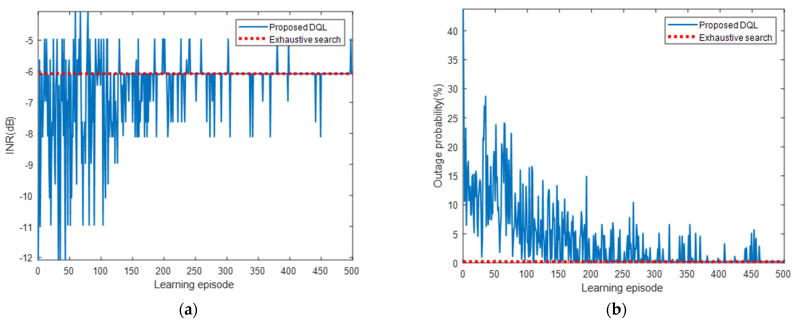
(**a**) INR and (**b**) $p_{out}$ for each learning episode for interfered receiver ②.

**Figure 14:**
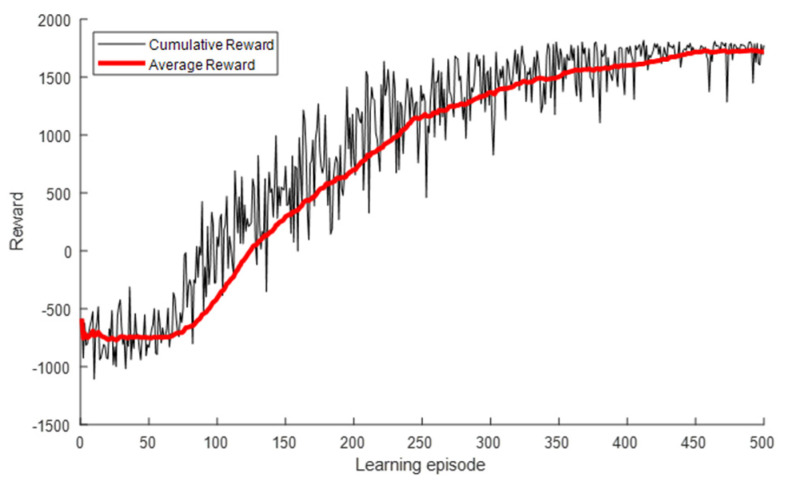
Reward for each learning episode for interfered receiver ②.

As shown in Figure 13, the INR and $p_{out}$ converged to the optimal values of the exhaustive search algorithm. Similar to the case of receiver ①, as the learning progressed, the INR converged from −12.08 to −6.08 dB, and the $p_{out}$ converged from 43.7% to 0.2%. Figure 14 shows that the reward gradually converged at approximately 300 episodes, indicating that the proposed DQL training process allowed the agent to quickly and stably learn the power control algorithm. We compared the learning results of the DQL and DDQL. Even when the DDQL was used, the results were the same as in Table 5 and Figure 13 and Figure 14, verifying that the desired learning is attainable with the DQL only.

#### 5.2.3. Simulation Results for Interfered Receiver ③

**Figure 15:**
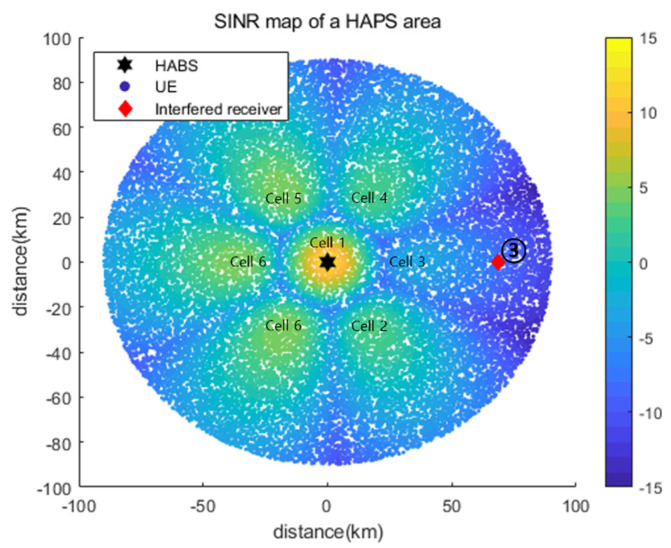
SINR map based on $P_{c}$ obtained using the proposed DQL-based power control algorithm for interfered receiver ③.

Figure 15 shows the SINR map based on $P_{c}$ obtained using the proposed DQL-based power control algorithm for interfered receiver ③. The interfered receiver was located in the azimuth main lobe direction of Cell_3. It was closer to the HAPS than the receiver considered in Section 5.2.1 and was more severely affected by Cell_3; $INR_{th}$ was not satisfied even for the minimum power of Cell_3. Thus, the optimal power control adjusted the power of Cell_2 and Cell_4, which caused the second-most interference. Table 6 presents a comparison of the $P^{*}$ values obtained using an exhaustive search and $P_{c}$ and a comparison of the DQL and DDQL results. Although the $p_{out}$ of $P_{c}$ was 0.6% higher than that of $P^{*}$, it corresponded to the third-smallest value among the 78,125 values generated by the exhaustive search algorithm. In summary, the proposed power control algorithm achieved outstanding performance close to the optimal value.

**Figure 16:**
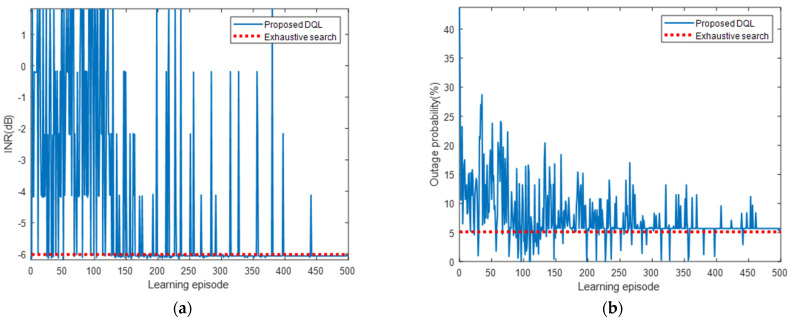
(**a**) INR and (**b**) *p_out_* for each learning episode for interfered receiver ③.

**Figure 17:**
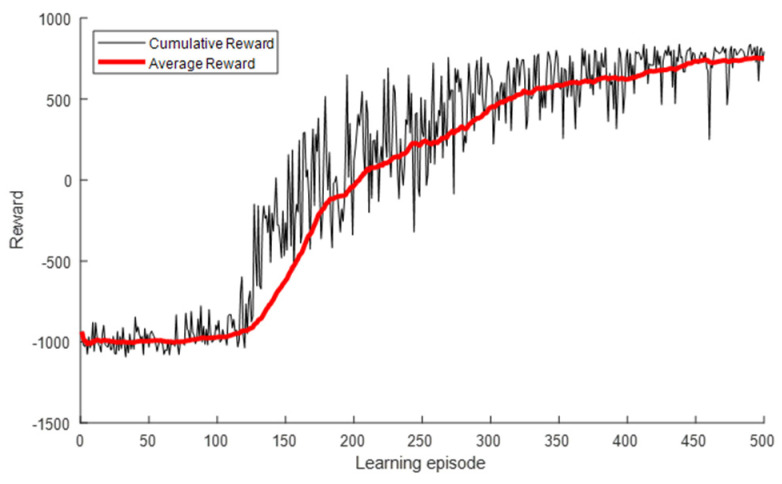
Reward for each learning episode for interfered receiver ③.

As shown in Figure 16, the INR and $p_{out}$ converged to the optimal values of the exhaustive search algorithm, with slight gaps. Similar to the results presented in Section 5.2.1, as the learning progressed, the INR converged from −6.19 to −6.06 dB, and the $p_{out}$ converged from 43.7% to 5.7%. Figure 17 shows the cumulative and average rewards for each learning episode. The reward exhibited no noticeable improvement until approximately 130 episodes, after which it rapidly increased and then gradually converged at approximately 350 episodes. This is because to satisfy the $INR_{th}$, more agents had to take action, and the actions had to be more diverse. Nonetheless, the proposed DQL training process allowed the agent to learn the power control algorithm quickly and stably. We compared the learning results of the DQL and DDQL. Even when the DDQL was used, the results were the same as in Table 6 and Figure 16 and Figure 17, verifying that the desired learning is attainable with the DQL only.

## 6. Conclusions

This paper proposed a DQL-based transmission power control algorithm for multicell HAPS communication that involved spectrum sharing with existing services. The proposed algorithm aimed to find a solution to the power control optimization problem for minimizing the outage probability of the HAPS downlink under the interference constraint to protect existing systems. We compared the solution with the optimal solution acquired using the exhaustive search algorithm. The simulation results confirmed that the proposed algorithm was comparable to the optimal exhaustive search.

Future work will include various power levels and expanding to multiple-HAPS communication in spectrum sharing with multiple interference systems. Since the increase in the power level could reveal a value-based algorithm’s limit, it is preferred to apply the policy-based algorithm. Given that multiple-HAPS communication could lead to the non-stationarity problem of multiagent reinforcement learning, its solution would be worth studying.

## Figures and Tables

**Table 1:** HAPS system parameters.

Parameter	Value
Center frequency (f)	2545 MHz
Channel bandwidth (BW)	20 MHz
Area radius	90 km
$\mathrm{Altitude}\;(h_{HAPS}$)	20 km
$\mathrm{Number}\;\mathrm{of}\;\mathrm{cells}\;(N_{cell}$)	7
Antenna pattern	Recommendation ITU-R M.2101
$\mathrm{Element}\;\mathrm{gain}\;(G_{E,max}$)	8 dBi
Horizontal/vertical 3 dB beamwidth of single element	65° for both H/V
Antenna array configuration (Row × column)	2 × 2 elements (1st layer cell) 4 × 2 elements (2nd layer cell)
$\mathrm{Ohmic}\;\mathrm{losses}\;(L_{ohm})$	2 dB
Antenna tilt	90° (1st layer cell) 23° (2nd layer cell)
Antenna polarization	Linear/±45°
$\mathrm{Number}\;\mathrm{of}\;\mathrm{distributed}\;\mathrm{UEs}\;(N_{UE}$)	1000
*UE* height	1.5 m
*UE* antenna gain	−3 dBi
$\mathrm{Minimum}\;\mathrm{required}\;\mathrm{SIN}R\;(\eta_{o}$)	−10 dB

**Table 2:** Interfered system (IMT BS) parameters.

Parameter	Value
Center frequency (f)	2545 MHz
Channel bandwidth (BW)	20 MHz
$\mathrm{Noise}\;\mathrm{figure}\;(N_{f})$	5 dB
$\mathrm{Antenna}\;\mathrm{height}\;(h_{V})$	20 m
Antenna tilt	10°
Antenna pattern	Recommendation ITU-R F.1336 (recommends 3.1) $k_{a}=0.7$ $k_{p}=0.7$ $k_{h\;}=0.7$ $k_{v}=0.3$ Horizontal 3 dB beamwidth: 65° Vertical 3 dB beamwidth is determined from the horizontal beamwidth equations in Recommendation ITU-R F.1336. Vertical beam widths of actual antennas may also be used when available.
Antenna polarization	Linear/±45°
$\mathrm{Maximum}\;\mathrm{antenna}\;\mathrm{gain}\;(G_{0})$	16 dBi
$\mathrm{Protection}\;\mathrm{criteria}\;(\;INR_{th}$)	−6 dB

**Table 3:** INR and $P_{out}$ for the interfered receiver locations.

Interfered Receiver	Location (km)	$INR\;\mathrm{for}\;P_{max}\;(\mathrm{dB})$	$INR\;\mathrm{for}\;P_{min}\;(\mathrm{dB})$	$P_{out}\;\mathrm{for}\;P_{max}\;(\%)$	$P_{out}\;\mathrm{for}\;P_{min}\;(\%)$
①	100, 0, 0.02	−3.01	−11.01	0	43.7
②	77.9, 45, 0.02	−4.08	−12.08	0	43.7
③	65.8, 0, 0.02	1.81	−6.19	0	43.7

**Table 4:** Performance comparison for interfered receiver ①.

	$P_{Cell\_1}\;(\mathrm{dBm})$	$P_{Cell\_2}\;(\mathrm{dBm})$	$P_{Cell\_3}\;(\mathrm{dBm})$	$P_{Cell\_4}\;(\mathrm{dBm})$	$P_{Cell\_5}\;(\mathrm{dBm})$	$P_{Cell\_6}\;(\mathrm{dBm})$	$P_{Cell\_7}\;(\mathrm{dBm})$	INR (dB)	$P_{out}$ (%)
Optimal	37	34	30	34	34	34	34	–6.93	0.6
DQL	37	34	30	34	34	34	34	–6.93	0.6
DDQL	37	34	30	34	34	34	34	-6.93	0.6

**Table 5:** Performance comparison for interfered receiver ②.

	$P_{Cell\;1}$ (dBm)	$P_{Cell\;2}$ (dBm)	$P_{Cell\;3}$ (dBm)	$P_{Cell\;4}\;(\mathrm{dBm})$	$P_{Cell\;5}\;(\mathrm{dBm})$	$P_{Cell\;6}\;(\mathrm{dBm})$	$P_{Cell\;7}\;(\mathrm{dBm})$	INR (dB)	$P_{out}$ (%)
Optimal	37	34	32	32	34	34	34	−6.08	0.2
DQL	37	34	32	32	34	34	34	−6.08	0.2
DDQL	37	34	32	32	34	34	34	−6.08	0.2

**Table 6:** Performance comparison for interfered receiver ③.

	$P_{Cell\;1}$ **(dBm)**	$P_{Cell\;2}$ **(dBm)**	$P_{Cell\;3}$ **(dBm)**	$P_{Cell\;4}\;(\mathrm{dBm})$	$P_{Cell\;5}\;(\mathrm{dBm})$	$P_{Cell\;6}\;(\mathrm{dBm})$	$P_{Cell\;7}\;(\mathrm{dBm})$	INR (dB)	$P_{out}$ (%)
Optimal	37	34	26	32	34	34	34	−6.02	5.1
DQL	37	32	26	32	34	34	34	−6.06	5.7
DDQL	37	32	26	32	34	34	34	−6.06	5.7

## Data Availability

Not applicable.
